# Survival of SARS-COV-2 under liquid medium, dry filter paper and acidic conditions

**DOI:** 10.1038/s41421-020-00191-9

**Published:** 2020-08-14

**Authors:** Zhiping Sun, Xia Cai, Chenjian Gu, Rong Zhang, Wendong Han, Yun Qian, Yuyan Wang, Wei Xu, Yang Wu, Xunjia Cheng, Zhenghong Yuan, Youhua Xie, Di Qu

**Affiliations:** 1 BSL-3 Laboratory of Fudan University, School of Basic Medical Sciences, Shanghai Medical College, Fudan University, Shanghai, China; 2 Key Laboratory of Medical Molecular Virology (MOE/NHC/CAMS), Department of Medical Microbiology and Parasitology, School of Basic Medical Sciences, Shanghai Medical College, Fudan University, Shanghai, China

**Keywords:** Cell biology, Molecular biology

Dear Editor,

The pneumonia caused by a novel coronavirus was first reported in December 2019 in Wuhan of China, and since then has become a pandemic^1,2^. International Committee on Taxonomy of Viruses (ICTV) named the virus as severe acute respiratory syndrome coronavirus 2 (SARS-CoV-2)^3^. SARS-COV-2 is transmitted mainly through respiratory droplets and close contact^4^. The fast spread of the coronavirus disease (COVID-19)^3^ suggests that SARS-COV-2 is highly contagious. The virus remained viable in the medium for 7 days at 22 °C and 1 day at 37 °C^5^. On dry surfaces at room temperature (RT), the virus was reported viable for 1 day on the surface of cloth, for 4 days on stainless steel, and for 7 days on the outer layer of a surgical mask, whereas no infectious virus was recovered from the surfaces of printing and tissue papers after a 3-h incubation^5^. Here, we first investigated the infectivity of SARS-COV-2 using a plaque-purified strain nCoV-SH01 isolated from a patient in Shanghai (GenBank MT121215)^6^, studied subsequently its stability in liquid medium, on dry filter paper, and under acidic condition (pH2.2) at RT. It would provide guidance on application appropriate measures to control the spread of SARS-COV-2 and improve laboratory biosafety management.

First the virus stock of nCoV-SH01 was quantified on Vero-E6 cells by plaque forming assay (plaque forming unit) and TCID_50_ assay (tissue culture infection dose), as 6 × 10^5^ PFU/mL and 2.8 × 10^6^ TCID_50_/mL, respectively. Cytopathic effects (CPE) appeared at 24 h post inoculation (h.p.i.) with 100–2000 PFU of the virus titer, at 48 h.p.i. with 5–50 PFU (Supplementary Fig. S1a), and at 72 h.p.i. with 1 PFU virus (Table 1a and Supplementary Fig. S1b). Based on that, we used 1.2 × 10^3^ PFU (3.75 Log_10_TCID_50_) virus in subsequent experiments.

**Table 1:** Infectivity of SARS-COV-2 at different virus titers.

a Vero-E6 cell infected by different PFU of the virus										
Day (d.p.i.)	Cells no virus	CPE induced by virus (×10^2^ PFU) inoculation^a^								
		20	10	5	2.5	1	0.5	0.1	0.05	0.01
1	–	++++	++++	++	+	−	−	−	−	−
2	–	/	++++	++++	++++	++++	++++	++++	++++	−
3	–	/	/	/	/	/	/	++++	++++	++++

b Stability of SARS-COV-2 in liquid medium at room temperature (RT)						
Day (d.p.i.)	Detection assay	Infectivity of 1.2 × 10^3^ PFU virus in liquid medium at RT^b^				
		Day 0	Day 1	Day 2	Day 3	Day 4
1	CPE	++	++	−	−	−
2	CPE	++++	++++	+~+++	+~++	−
	Log_10_TCID_50_	3.75 ± 0.43	2.92 ± 0.29	2.17 ± 0.14	1.35 ± 0.33	UD
	RT-PCR ORF1ab	15.61 ± 0.28	15.64 ± 0.09	15.74 ± 1.13	33.36 ± 1.05	UD
	N	15.99 ± 0.10	15.66 ± 0.04	16.27 ± 0.06	32.92 ± 1.33	UD
3	CPE	/	/	++++	++~+++	−
4	CPE	/	/	/	+++	−
5	CPE	/	/	/	+++	−
	RT-PCR	ND	ND	ND	ND	UD

c Stability of SARS-COV-2 on dry filter paper at room temperature (RT)						
Day (d.p.i.)	Detection assay	Infectivity of 1.2 × 10^3^ PFU virus on dry filter paper at RT^b^				
		Day 0	Day 1	Day 2	Day 3	Day 4
1	CPE	−	±	−	−	−
2	CPE	+ + + +	+	−~+	±	−
	Log_10_TCID_50_	3.42 ± 0.13	2.25 ± 0.25	2.08 ± 0.14	UD	UD
	RT-PCR ORF1ab	15.25 ± 0.22	29.05 ± 0.45	35.09 ± 0.18	35.74 ± 0.09	UD
	N	15.06 ± 0.59	28.60 ± 0.15	35.12 ± 0.42	35.98 ± 0.19	UD
3	CPE	/	+	+	±	−
4	CPE	/	+~++++	+	±	−
5	CPE	/	++~++++	+~++	++	−
	RT-PCR	ND	ND	ND	ND	UD

d Effect of pH 2.2 saline on the survival of SARS-COV-2													
Day (d.p.i.)	CPE induced by the virus after incubation in pH 2.2 saline^c^											Virus control (pH 7.12) 60 min	
	Incubation for 30 s (×10^2^ PFU)							30 min (×10^2^ PFU)		60 min (×10^2^ PFU)			
	12	10	5	1	0.2	0.05	0.01	12	10	12	10	12	0.01
1	+++	++	−	−	−	−	−	−	−	−	−	++++	−
2	++++	++++	++++	−	−	−	−	++++	−	++++	−	++++	−
3	/	/	/	++++	++++	++++	−	/	−	/	−	/	++++
4	/	/	/	/	/	++++	−	/	−	/	−	/	/

d.p.i.: days post-inoculation. CPE of Vero E6 cells was checked under microscope. Degree of CPE, “++++”, >75% of cells; “+++”, 50–75%; “++”, 25–50%; “+”, 0–25%; “±”, not clear-cut; “−”, no CPE. UD under detectable level, ND not determined.

^a^The experiments were carried out in triplicate wells for each dilution. The cytopathic effects were observed under a microscope daily (Supplementary materials).

^b^The Viral RNA in the supernatant after the 2-day and 5-day incubation was extracted. Quantitative RT-PCR was performed using the primers and probes for viral ORF1ab and N. Ct values were presented as mean ± SD. TCID_50_ was calculated by Karber method (Supplementary materials).

^c^The experiments were carried out in triplicate wells for each dilution. The virus control was treated with physiological saline (final pH = 7.12) for 60 min. Physiological saline (pH = 7.0) was used as a blank control. The cytopathic effects were observed under a microscope daily (Supplementary materials).

SARS-COV-2 can be shed into wet or dry surrounding by droplets or aerosol^4^. How stable is the virus in different environment? We first determined viral stability in liquid medium. 1.2 × 10^3^ PFU (3.75 Log_10_TCID_50_) virus in DMEM was added into each well kept in a wet box at RT. After set for 1, 2, 3, 4, 5, 6, or 7 days, respectively, 100 μL of the virus solution was transferred from each sample onto Vero-E6 monolayer. CPE were checked daily till day 5. We found that when the virus had been kept in the medium at RT for 1 day, CPE appeared at 24 h.p.i., which was like the untreated virus control. When the virus had been kept for 2 or 3 days, CPE emerged at 48 h.p.i. (Table 1b). By day 3, the virus titer decreased 2 Logs (from 3.75 to 1.35 Log_10_TCID_50_). When the virus had been left in the medium for more than 4 days, no CPE was observed. The loss of infectivity was confirmed by TCID_50_ assay, immune florescence staining with the antiserum against viral N protein (Supplementary Fig. S2a) and qRT-PCR (Ct value over the cutoff >38, Supplementary Table S1). We then investigated viral stability on dry filter paper at RT. 1.2 × 10^3^ PFU (3.75 Log_10_TCID_50_) virus in 5 μL DMEM was added onto sterilized filter paper in plates. After completely dried, the plates were put into a dry box at RT. After set for 1, 2, 3, 4, 5, 6, or 7 days, the virus on the filter paper was eluted with DMEM, respectively. The eluted virus titer was 3.42 Log_10_TCID_50_ after the virus remained on the paper air dried for 1 h (recovery efficiency was 10^3.42^/10^3.75^ = 10^−0.33^ = 46.77%) and CPE appeared on day 2 post inoculation. When the virus had been kept on dried filter paper for 1 or 2 days at RT, CPE appeared on day 4 or 5 post inoculation, respectively (Table 1c), and the virus titer dropped to 2.17 Log_10_TCID_50_ with the 2-day incubation. For the 3-day incubation at RT, CPE appeared at day 5 post inoculation but the virus titer could not be determined by TCID_50_ assay, and for the 4-day incubation, no CPE was observed. The loss of infectivity was also confirmed by immune florescence staining with the anti-N serum (Supplementary Fig. S2b) and qRT-PCR. Our results show that COVID-19 virus can survive for 3 days in liquid medium or on dry filter paper. For the 3-day incubation in liquid medium at RT, viable virus left only 1.35 Log_10_TCID_50_ (initial titer was 3.75). For the 3-day incubation on dry filter paper at RT, although CPE was observed, the survived virus could not be quantified by TCID_50_ assay. The loss of virus viability in prolonged incubation (>4 days) was confirmed by N protein immunofluorescence staining, qRT-PCR, and further verified by blind passage of the supernatant for three generations. In Alex’s study, the virus remained viable in the medium for 7 days at 22 °C^5^. The reason for the longer survival time in their report might be the higher viral titer they used (~6.7 Log_10_TCID_50_ versus 3.75 Log_10_TCID_50_ in this study). Regardless, our results show that SARS-COV-2 is highly infectious and relatively stable in the environment, which underscores the importance of environmental disinfection and hand hygiene.

Since some coronaviruses can cause infectious diseases of digestive tract via gastrointestinal transmission, such as mouse hepatitis virus (MHV), porcine epidemic diarrhea virus (PEDV), and feline enteric coronavirus (FECV)^7–9^. Bioinformatics analysis of single-cell transcriptomes revealed that ACE2 was expressed in esophagus squamous epithelium cells and enterocytes of ileum and colon^10^, hence SARS-COV-2 might be potentially transmitted via the fecal-oral route^11,12^. We speculate that SARS-COV-2 would have to survive the gastric acidic environment if the virus is indeed transmitted via the fecal-oral route. Consequently, we determined the survival of SARS-COV-2 under acidic condition in vitro. Various amount of the virus (1.2 × 10^3^, 1.0 × 10^3^, 5 × 10^2^, 1 × 10^2^, 0.2 × 10^2^, 0.05 × 10^2^, 0.01 × 10^2^ PFU) was treated with acidic physiological saline (pH 2.2) at RT for 30 s, 30 min or 60 min, respectively. After treatment, each viral sample was adjusted to pH 7.28 and added onto Vero-E6 monolayer. As shown in Table 1d, after a 30-s incubation in pH 2.2 saline, significant CPE appeared at 48 h.p.i. with 1.2 × 10^3^, 1.0 × 10^3^, 5 × 10^2^, or 1 × 10^2^ PFU of the virus. CPE appeared at 72 or 96 h.p.i. with lower virus titers (1 × 10^2^, 0.2 × 10^2^, or 0.05 × 10^2^ PFU). No CPE was seen with 0.01 × 10^2^ PFU virus while CPE from the same amount as the virus control (0.01 × 10^2^ PFU) was readily observed at 72 h.p.i. After the 30-min or 60-min incubation in pH 2.2 saline, no CPE was observed with the virus titers equal and below 1.0 × 10^3^ PFU, whereas CPE appeared with 1.2 × 10^3^ PFU virus although the survived virus could not be determined by TCID_50_ assay (Table 1d and Supplementary Fig. S3) and confirmed by immune florescence staining (Supplementary Fig. S4). Transmission of SARS-COV-2 through the fecal-oral route is currently uncertain. Although SARS-COV-2 RNA has been detected in patients’ stool^11,13,14^, infectious virus was not readily isolated from stool. In the present study, when 1.2 × 10^3^ PFU virus was treated with the acidic saline of pH 2.2 for 30 or 60 min, virus survival could be observed as manifested by CPE but failed to be quantified, whereas no apparent virus survival was detected with lower virus titers (<1.0 × 10^3^ PFU) treated under the same acidic condition. The results suggest that SARS-COV-2 at a certain high titer might survive the acidic environment of the stomach for a certain period. Although it is unclear whether the virus can replicate in the intestine, the survived virus in the gastrointestinal tract may be excreted in faeces, which would indicate the importance of stool disinfection. Whether the virus can be transmitted through the fecal-oral route needs further study.

In conclusion, our findings show that SARS-COV-2 can survive for 3 days in liquid medium or on dry filter paper, and the virus at a high titer can survive under acidic condition that mimics the gastric environment. Our study would provide guidance on application of appropriate measures to control the spread of SARS-COV-2 and improve laboratory biosafety.

## Supplementary information

Supplementary Materials, Table and Figures

## Supplementary information

**Supplementary Information** accompanies the paper at (10.1038/s41421-020-00191-9).
