# Supplementary material for: Survival of SARS-COV-2 under liquid medium, dry filter paper and acidic conditions
Source: Cell Discov. 2020 Aug 14;6:57. doi: 10.1038/s41421-020-00191-9 (PMC7426817; doi:10.1038/s41421-020-00191-9)
Supplement: Supplementary file 1 — Supplementary Materials, Table and Figures [file 41421_2020_191_MOESM1_ESM.pdf]

Supplementary Material

Material and Methods

Viral strain

The COVID-19 virus (strain nCoV-SH01, SARS-CoV-2/human/CHN/SH01/2020, GenBank accession no. MT121215) was isolated from the nasal-pharyngeal swab of a clinically confirmed COVID-19 patient in Shanghai. The virus was plaque-purified and the viral stock (6×10^5 PFU/mL, Plaque Forming Unit) was prepared, aliquot and stored at -80°C. All the viral experiments were carried out in the biosafety level 3 laboratory of Fudan University.

Cell culture

Vero-E6 cells were maintained in DMEM containing 4 mM glutamine, 110 mg/L sodium pyruvate, and 4.5g/L glucose (Corning cellgro, Manassas, USA) supplemented with penicillin (100 IU/ mL), streptomycin (100 μg/mL) (Corning cellgro, Manassas, USA), and 10% fetal bovine serum (FBS, Corning, USA). Vero-E6 cells were seeded into the 24-well plates (Corning costar, USA) at a density of 1.0×10^5 cells/well and incubated for 24 hours till 80% confluence for viral infection.

Viral infection

Vero-E6 cells in 24-well plates were washed twice with 500 μL PBS per well. The nCoV-SH01 stock was diluted to different virus concentrations with DMEM containing 5 μg/mL trypsin (Sigma, St Louis, USA). 100 μL of the diluted viral solution was added into each well and incubated under 5% CO_2 at 37°C for 1 hour. After the viral inoculum was aspirated, 500 μL of cell maintenance medium (DMEM supplemented with 10% FBS and antibiotics) was added to each well and cultured in a humidified chamber with 5% CO_2 at 37°C. The cytopathic effect (CPE) was checked daily under a microscope (EVOS, Thermo Fisher, USA).

$TCID_{50}$ determination of the COVID-19 virus

The virus was serially 10-fold diluted by DMEM and of each dilution was added to four wells (100uL/well) with VeroE6 cells (96-well plate, 3.0×10^4cell/well). Cells were cultured at 37°C with 5% CO_2 for 72 hours, the cytopathic effect in each well were recorded. The virus $TCID_{50}$ was calculated by Karber method. The assay was done independently three times.

Survival of the COVID-19 virus under liquid medium condition

The nCoV-SH01 1.2×10^3 PFU in 200 μL DMEM was added into each well of 48-well

plates (Corning costar, USA). The plates were put in a wet box (relative humility ~80%) at room temperature (~22°C). After 1, 2, 3, 4, 5, 6, or 7 days, 100 μL was transferred from each well onto Vero-E6 monolayer in 24-well plates supplemented with 400 μL/well of cell maintenance medium containing 5 μg/mL trypsin. The plates were incubated in the humidified chamber with 5% CO_2 at 37°C and the cytopathic effects were checked daily with a microscope for 5 days. The survived virus determined by $TCID_{50}$.

Survival of the COVID-19 virus on dry filter paper

The nCoV-SH01 1.2×10^3 PFU in 5 μL DMEM was added dropwise onto sterilized filter paper (5 mm × 5 mm) in each well of 48-well plates. When the filter paper was completely dry, the plates were put into a dry box and kept at room temperature in a biosafety cabinet. After 1, 2, 3, 4, 5, 6, or 7 days, the filter paper was washed with 100 uL of DMEM and the elute was added onto Vero-E6 monolayer in 24-well plates supplemented with 400 μL/well of cell maintenance medium containing 5 μg/mL trypsin. The plates were incubated in the humidified chamber with 5% CO_2 at 37°C and the cytopathic effects were checked daily with a microscope for 5 days. The survived virus and recovery efficiency of virus eluted from the dry filter paper were determined by $TCID_{50}$. The recovery efficiency of virus was calculated by virus titer eluted from dry filter paper (3.42 $Log_{10}TCID_{50}$) divided by the initiate viral titer (3.75 $Log_{10}TCID_{50}$).

Survival of the COVID-19 virus under acidic condition

2.4×10^3 PFU of nCoV-SH01 in 20 μL DMEM was added to 180 μL of pH2.0 physiological saline (final pH 2.2) and kept at room temperature (~22°C) for 30 seconds, 30 minutes and 60 minutes respectively. Various amount of acid-treated virus (1.2×10^3, 1.0×10^3, 5×10^2, 1×10^2, 0.2×10^2, 0.05×10^2 or 0.01×10^2 PFU) was diluted with DMEM to 100 μL and added onto Vero-E6 monolayer cultured in 24-well plates with 400 μL/well of cell maintenance medium containing 5 μg/mL trypsin (final pH ~7.28). The virus controls (1.2×10^3 and 0.01×10^2 PFU) were prepared in physiological saline (pH7.0) and incubated for 60 minutes. The blank control is physiological saline (pH7.0). The plates were incubated in the humidified chamber with 5% CO_2 at 37°C and the cytopathic effects were checked daily with a microscope. The survived virus determined by $TCID_{50}$.

Detection of the COVID-19 virus with RT-PCR

Viral RNA was extracted and purified by QIAamp MinElute Virus Spin Kit (Qiagen, Hilden, Germany) following the instruction of manufacturer. 100 μl of cultured supernatant was used for each extraction and viral RNA was eluted in 50 μL of RNase-free water. The purified viral RNA was detected with qRT-PCR using a commercial kit

(BioGerm Shanghai Berger Medical Technology Co., Ltd.) following the instruction of manufacturer. Briefly, 12 μL of qRT-PCR reaction solution, 4 μL of qRT-PCR enzyme mixture (Reverse transcriptase, RNA enzyme inhibitor, Taq enzyme), 4 μL of primer probe (2019-nCoV ORF1ab/N), and 5 uL of viral RNA were added into each well of a 96-well plate (Opical 96-Well Reaction Plate, Applied Biosystems, USA) to a final volume of 25 μL. The plate was membrane-sealed, centrifuged at low speed and placed in a real time PCR machine (ABI7500, Applied Biosystems, USA). The amplification/detection included a reverse transcription step at 50℃ for 10 minutes, a pre-denaturation step at 95℃ for 5minutes, and a cycling program of denaturing at 95℃ for 10 seconds and annealing/extension/fluorescence detection at 55℃ for 40 seconds for 40 cycles (FAM and VIC channels for the ORF1ab and N probes, respectively). Ct value> 38 was taken as negative cutoff. The primers and probes used were listed in Supplementary Table S1.

COVID-19 virus infected cells detected with immunofluorescence

The nucleocapsid (N) protein gene was reversed transcribed from the RNA genome of nCoV-SH01 and inserted into a prokaryotic expression plasmid (pET-28). The purified recombinant N protein was mixed with adjuvant AD20Gold+ (Beijing Aidi Weixin Biotechnology Co., Ltd., Beijing, China). BABL/c mice were immunized by intramuscular injection with priming and boosting at an interval of 14 days. The anti-sera from immunized mice were collected at day 28 after immunization.

The virus-infected cells were washed with precooled PBS and fixed with 4% paraformaldehyde (Sigma, St Louis, USA) for 10-15 min at RT, and then treated with 1% Triton X-100 (Sigma, St Louis, USA) for 10 min at RT. After blocked with 3% BSA (Meilun, Shanghai, China) for 1 hour at 37˚C, 1:500 diluted mouse anti-N serum was added into the cells and incubated at 4˚C overnight. After blocked with 3% BSA, 1:200 FITC labeled donkey anti-mouse IgG (H+L, highly cross-adsorbed secondary antibody, Invitrogen, Alexa Fluor 488) were added on the cells and incubated for 1 hour at 37˚C. The cells were stained with DAPI (Sangon, Shanghai, China) at RT for 5-10 min, and observed under microscope.

Supplementary Table S1. The primers and probes used for the detection of the COVID-19 virus

Target gene	Sequences
ORF1ab gene	
Forward primer	5'-CCCTGTGGGTTTTACACTTAA-3'
Revered primer	5'-ACGATTGTGCATCAGCTGA-3'
Fluorescent probe	5'-FAM-CCGTCTGCGGTATGTGGAAAGGTTATGG-BHQ1-3'
N gene	
Forward primer	5'-GGGGAACTTCTCCTGCTAGAAT-3'
Revered primer	5'-CAGACATTTTGCTCTCAAGCTG-3'
Fluorescent probe	5'-VIC-TTGCTGCTGCTTGACAGATT-TAMRA-3'

Supplementary Figure

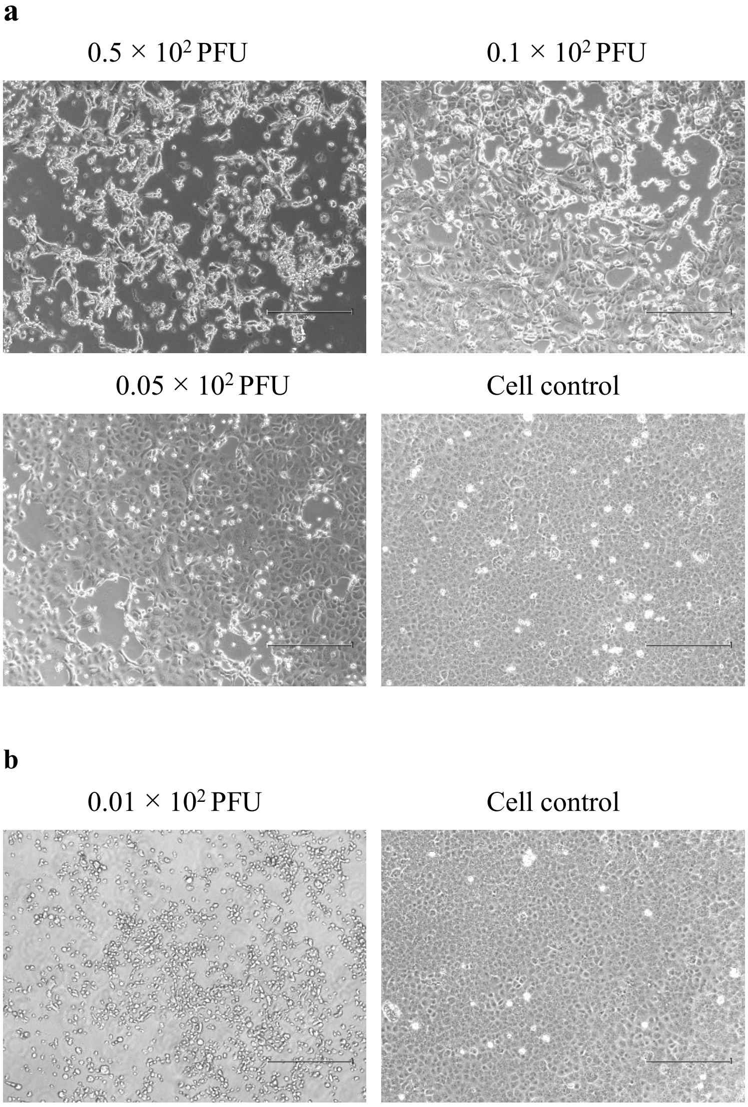

Supplementary Fig. S1 The cytopathic effects resulted from the infection of Vero-E6 cells by different titers of the COVID-19 virus. a CPE at day 2 after viral inoculation. **b** CPE at day 3 after viral inoculation. The nCoV-SH01 stock was diluted to different virus concentrations. 100 μL of the diluted viral solution was added into each well and incubated under 5% CO_2 at 37°C for 1 hour. After the viral inoculum was aspirated, 500 μL of cell maintenance medium was added to each well and cultured in a humidified chamber with 5% CO_2 at 37°C. The cytopathic effects were observed under a microscope. The experiments were carried out in triplicate wells for each dilution. The cell control was without the virus inoculation. CPEs resulted from infection with low concentrations of the virus were presented.

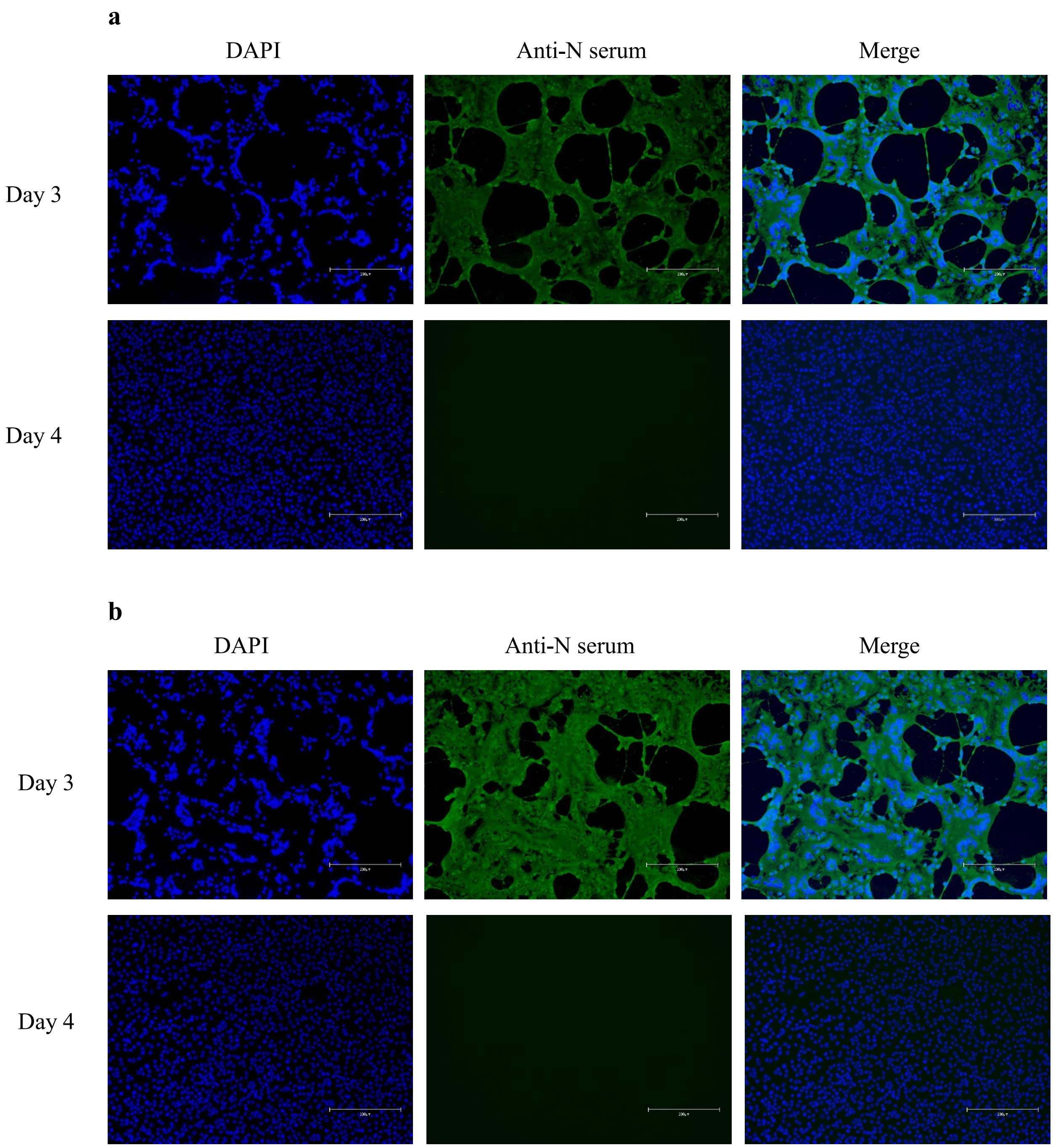

Supplementary Fig. S2 Detection of infections with the COVID-19 virus by immunofluorescence with anti-N serum. a Vero-E6 cells inoculated with virus in liquid condition for 3 or 4 days. **b** Vero-E6 cells inoculated with virus on dry filter paper for 3 or 4 days. Virus (1.2×10^{3}PFU) was incubated in liquid condition (**a**) or on dry filter paper (**b**) for 3 or 4 days, and then inoculated onto Vero-E6 cells respectively. At day 2 post viral infection, the cells were fixed and stained for viral N protein using mouse anti-N serum (1:500 dilution) and FITC labeled donkey anti-mouse IgG (1:200 dilution) (green). The cell nuclei was stained with DAPI (blue). The viral N proteins were observed in the cells inoculated with the virus after a 3-day incubation under both wet and dry conditions but not with the virus after a 4-day incubation.

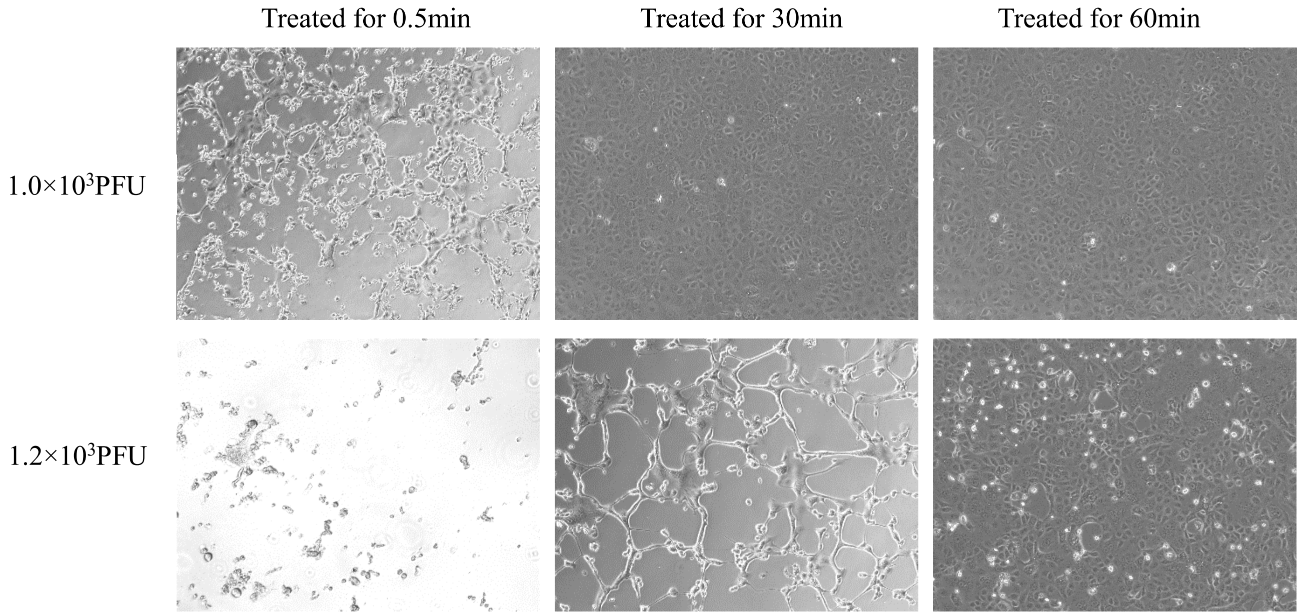

Supplementary Fig. S3 The cytopathic effects resulted from the infection of Vero-E6 cells by the COVID-19 virus under acidic condition for minutes

The nCoV-SH01 at 1.0×10^3PFU or 1.2×10^3PFU were treated with pH2.2 saline for 0.5, 30 or 60 minutes, and then inoculated onto Vero-E6 cells, respectively. At day 2, the cytopathic effects were observed under a microscope. The experiments were carried out in triplicate wells for each dilution.

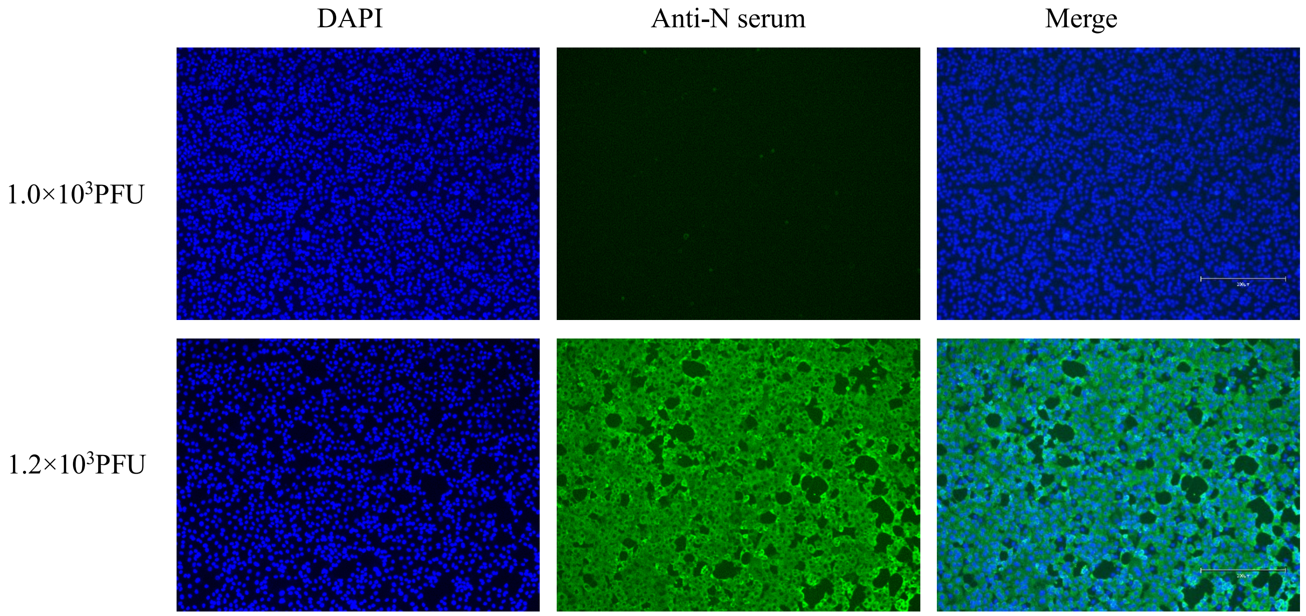

Supplementary Fig. S4 Stability of the COVID-19 virus under acidic condition by immunofluorescence with anti-N serum

Different titers of nCoV-SH01 (1.0×10^3PFU or 1.2×10^3PFU) were treated with pH2.2 saline for 60 minutes and then inoculated onto Vero-E6 cells respectively. At day 2 post viral infection, the cells were fixed and stained for viral N protein using mouse anti-N serum (1:500 dilution) and FITC labeled donkey anti-mouse IgG (1:200 dilution) (green). The cell nuclei were stained with DAPI (blue).
